# Supplementary material for: Vulnerability in maternal, new-born, and child health in low- and middle-income countries: Findings from a scoping review
Source: PLoS One. 2022 Nov 11;17(11):e0276747. doi: 10.1371/journal.pone.0276747 (PMC9651566; doi:10.1371/journal.pone.0276747)
Supplement: S1 Table — (PDF) [file pone.0276747.s001.pdf]

S1 Table: Characteristics of included studies on vulnerabilities in newborn and child health

Author & year	Country	Research method	Study design	Study objectives
Akseer 2019	Afghanistan	Quantitative	Observational study	To evaluate associations of conflict severity with improvement of health system performance, use of health services, and child nutrition outcomes in Afghanistan during the 2003 to 2018 reconstruction period.
Ali Hirani 2014	Pakistan	Qualitative	Literature review	This paper reports on the issues, challenges and way forward of internally displaced children in disaster relief camps in Pakistan who are vulnerable in all aspects of their health i.e. mental, physical, social and spiritual.
Andreani 2006	Brazil	Quantitative	Observational study	To provide a panorama on premature birth and its repercussions in the familiar scope, showing the vulnerability of the baby in its physical and affective development, as well as the emotional crisis that the parents go through during this event, besides the alteration in the family dynamics.
Biemba 2013	Mozambique	Quantitative	Observational study	To examine specifically the role of orphanhood and OVC status in predicting child outcome in health, education, access to food and nutritional status.
Chiao (2020)	Regions: Africa, Eastern Europe, Central Asia, South East Asia, Latin America	Quantitative	Observational study	To investigate whether the presence of symptoms of acute respiratory infection (ARI) are contingent on environmental vulnerabilities such as the region, the level of ambient air pollution, community orphanhood status, and household poverty among young children under five.
Chiao 2017	Democratic Republic of Congo, Malawi and Nigeria	Quantitative	Observational study	To examine the linkages between children's vulnerability and ARI symptoms, with a focus on the individual as well as community levels.
Davis 1996	Tanzania, Uganda and Democratic Republic of Congo	Quantitative	Observational study	To compare the relative risk of death for young children with that for older children and adults under normal conditions and in emergency settings.
[1]Desclaux 2013	Senegal	Qualitative	Observational study	To analyze the emergence of a situation of economic vulnerability generated by a public health policy that is nevertheless globally appropriate in terms of accessibility and epidemiological effectiveness.
Dogan 2009	Turkey	Quantitative	Observational study	To adapt and adopt practices that have been advised for pediatricians to prevent ‘the vulnerable child syndrome’, information is needed also on the prevalence and correlates of perceived vulnerability in children in LMICs.
Drachler 2014	Brazil	Quantitative	Observational study	To develop and validate of a Social Vulnerability Index (SVI) including five social determinants of risk to health and demonstrates its application in the financing of primary care by the Unified Health System (SUS) in the State of Rio Grande do Sul.
Gaillard 2002	Kenya	Quantitative	Observational study	To examine intervention study to reduce mother-to-child transmission of HIV in Mombasa, Kenya.
Ghosh-Jerath 2013	India	Quantitative	Observational study	To assess the nutritional status of Sahariya tribes of Madhya Pradesh (MP), India.
Huls 2020	South Africa	Quantitative	Observational study	To examine associations of exposure to indoor particulate matter with a 50% cut-off aerodynamic diameter of 10 μm (PM10) and maternal smoking with infant lung function and the role of gene-environment interactions.

Author & year	Country	Research method	Study design	Study objectives
Johnson 2010	Rwanda	Quantitative	Observational study	To examine how the complex relationships between and within the proximate and socio-economic determinants mediate the poor health outcomes of children through their OVC status.
Kalibala 2012	Uganda	Mixed-method research	–	To engage national stakeholders in a priority-setting exercise to develop a scoring system to assess dimensions of children's vulnerability.
Kaye 2014	Uganda	Qualitative	Observational study	To gain an understanding of how obstetric complications affect the lives and livelihoods of survivors.
Lara-Valencia 2012	Mexico	Quantitative	Observational study	To explore the impact of contextual variables at the neighborhood level on a health marker in the city of Hermosillo, Mexico and discusses the importance of collaboration between planners and health professional to minimize the negative effect of contextual factors on urban health.
Lei 1999	Brazil	Quantitative	Observational study	To estimate the risk of linear growth retardation during the first two years of life as a result of household social vulnerability.
Mannan 2011	Bangladesh	Quantitative	Observational study	To assess the association of very severe disease (VSD) in newborns in rural communities with temperature, rainfall, and humidity.
Murray 2015	Brazil	Quantitative	Observational study	To test for sex differences in the association between fetal growth indices and attention problems at age four, in a large, prospective birth cohort from a middle-income country.
Ncube 2016	Botswana	Qualitative	–	To explore and describe the lived experiences of mothers regarding care of their hospitalized preterm infants in a neonatal unit in a public hospital in Gaborone, Botswana.
Noorbakhsh 2019	Iran	Quantitative	Observational study	To assess the relation of serum level of zinc, and Vitamins A and D with UTI in children with proven UTI.
Oliveira 2019	Brazil	Quantitative	Observational study	To verify the occurrence of the risk and protective factors for sudden infant death syndrome during nursing consultation.
Otani 2018	Mongolia	Quantitative	Observational study	To evaluate the health risks for children during the Dzud using various factors, including medical resources.
Pedraza 2016	Brazil	Quantitative	Observational study	To evaluate the nutritional status of under-five-year-old children living in a locality intentionally studied because of its social vulnerability and association with personal and socioeconomic characteristics.
Pollitt 1996	Guatemala	Quantitative	Implementation science	To test whether the timing of these nutritional events partially determines the nature and scope of the adverse effects and examine whether the timing of the measurement of a functional outcome (e.g., attention) determines the magnitude of the cognitive delay observed. Timing, therefore, is considered for both the predictor and the outcome.
Sartorius 2010	South Africa	Quantitative	Observational study	To assess changes in infant mortality patterns between 1992-2007 and to identify factors associated with infant mortality risk in the Agincourt sub-district, rural northeast South Africa.
Sartorius 2011	South Africa	Quantitative	Observational study	To assess temporal changes in child mortality patterns within the study site between 1992 and 2007, and to produce all-cause and cause-specific mortality maps to identify high risk

Author & year	Country	Research method	Study design	Study objectives
				areas.
Seidel 2000	South Africa	–	Observational study	To study the experiences of breastfeeding and vulnerability among a group of HIV-positive women in Durban, South Africa.
Siekmans 2014	Ghana, Malawi and Tanzania	Quantitative	Implementation science	To estimate the association between hemoglobin and immediate, underlying and basic causes of child anemia and variation in this association between years.
[1]Sochas 2017	Sierra Leone	Quantitative	Observational study	To quantify the extent of the drop in utilization of essential reproductive, maternal and neonatal health services in Sierra Leone during the Ebola outbreak by using interrupted time-series regression to analyze Health Management Information System (HMIS) data.
Thorne-Lyman 2018	Nepal	Quantitative	Observational study	(1) Assess the change in prevalence of child stunting and wasting in a post hoc sample of areas classified by the government as "earthquake-affected" from 2014 to 2016; (2) Examine in greater detail the shocks experienced by households, potential risk factors for malnutrition, and indicators of household resilience in the same sample.
Tudge 2013	Kenya and Brazil	Qualitative	Observational study	To compare heterogeneity of young children's vulnerability experiences in Kenya and Brazil.
[1]Ventura 2008	Brazil	Quantitative	Observational study	To investigate vulnerability and the consequent differences in access to health services and occurrences of deaths among infants under one year of age in the municipality of Embu.
Wang 2019	China	Quantitative	Observational study	To explore child neglect among preschool children in rural China and examine its association with social living ability
Winch 2005	Bangladesh	Qualitative	Observational study	To describe the organization of the neonatal period in Sylhet District, Bangladesh, the perceived threats to the well-being of neonates, and the ways in which families seek to protect them.
Worku 2017	Ethiopia	Quantitative	Observational study	To investigate the developmental performances of children between 3.5 and 71.8 months of age living in the SOS village of Jimma compared to age- and sex-matched family-reared children and to compare the social and emotional behavior of SOS to the family-reared children.
Yuan 2020	Guinea-Bissau, Guinea, Sierra Leone, Liberia, Cote d'Ivoire, Ghana, Togo, Benin, Nigeria, and Cameroon	Quantitative	Observational study	To identify which factor of social vulnerability predominantly affects infant mortality.
Yusuf 2010	Nigeria	Quantitative	Observational study	To investigate the relationship between the prevalence of childhood fever and socioeconomic factors including poverty in Nigeria, and to examine these effects at the regional levels.
Zakayo 2020	Kenya	Qualitative	Observational study	To examine vulnerability and agency across treatment-seeking journeys for acutely ill children and considered the implications for policy and practice.

[1] Study focused on vulnerability in maternal and child health.
