# Supplementary material for: Vulnerability in maternal, new-born, and child health in low- and middle-income countries: Findings from a scoping review
Source: PLoS One. 2022 Nov 11;17(11):e0276747. doi: 10.1371/journal.pone.0276747 (PMC9651566; doi:10.1371/journal.pone.0276747)
Supplement: S2 Table — (PDF) [file pone.0276747.s002.pdf]

S2 Table: Definitions and indices of vulnerability in new-born and child health

Author & year	Context	Definition by authors	Vulnerability indices	Vulnerability scale
Akseer 2019	Maternal health, child health	The authors defined vulnerability as those exposure to intense conflict and associated with worse maternal and child health outcomes.		-
Andreani 2006	Child health	Vulnerability was defined in relation to preterm birth: showing the baby's vulnerability in its physical and affective development.	–	-
Biemba 2013	Child health	Vulnerability was defined in relation to various markers, which, when suboptimal, makes a child vulnerable.<br>Markers of vulnerability are household wealth status, education levels of caregivers, living arrangements, relationship to head of household, orphanhood, and OVC status.	–	-
Chiao 2020	Child health	-	- Community orphanhood<br>- Household poverty<br>- Household smoking.	-
Chiao 2017	Child health	Vulnerable children were identified as those who had experienced the death of a family member who had been ill for at least three months during the past 12 months or who were a member of a household with a member who had been ill for at least three months during the past 12 months.	Community vulnerability<br>- Community OVC (Average prevalence of OVC aged under 18 years within a community cluster)<br>- Community sexual violence by male partners (Average prevalence of lifetime sexual violence by male partners against women aged 15–49 years within a community cluster).	-
Davis 1996	Child health	No specific definition of vulnerability was given by the authors.	Crude mortality rate (CMR).	-
Desclaux 2013	Maternal health, child health	No specific definition of vulnerability was given by the authors.	Indebtedness with no certainty for repayment.	-
Dogan 2009	Child health	The authors defined vulnerability as susceptibility of a child to life-threatening illnesses or death.	-	Child vulnerability Scale modification
Drachler 2014	Child health	No specific definition of vulnerability was given by the authors.	- Infant mortality<br>- Hospitalisation for conditions treatable by primary care.	-

Author & year	Context	Definition by authors	Vulnerability indices	Vulnerability scale
Johnson 2010	Child health	No specific definition of vulnerability was given by the authors.	Vulnerability status was measured in this study using the global definition of OVC status. That is children who faced:<br>- Loss of one or both parents<br>- Chronic illness in one or both parents for at least three months within the past 12 months.<br>- Living in a household with at least one chronically ill adult (18-59 years old) for at least three months within the preceding 12 months<br>- Living in a household where at least one adult (18-59 years old) had died in the past 12 months after chronic illness of at least three months.	-
Kalibala 2012	Child health	No specific definition of vulnerability was given by the authors	Details of indices are separately presented in Table 10 of the report.	-
Kaye 2014	Child health	'…exposure to increased health risk and health demands in the context of limited social and economic resources needed for protection against risks or mitigation of resultant social and economic consequences'.	–	-
Lara-Valencia 2012	Child health	Vulnerability was defined in terms of risk and risk exposure on the one hand and adaptive behaviours on the other.	A composite Built Environment Vulnerability Index (BEVI) aggregated by area unit was derived to characterise the spatial pattern of contextual factors.	-
Lei 1999	Child health	Vulnerability in this study was used in the following context: 'body size or growth retardation in children that together is likely to increase children's susceptibility to infections and have poor cognitive development'.	-	-
Mannan 2011	Child health	Conceptualised vulnerability in terms of age - new-borns are likely to be more vulnerable than older infants and adults.	–	
Ncube 2016	Child health	Vulnerability was conceptualised using preterm delivery and low birth weight (LBW).<br><br>*LBW and preterm infants are unable to regulate their body temperature and are vulnerable to numerous complications, including respiratory problems, neonatal jaundice, feeding problems and neonatal	–	-

Author & year	Context	Definition by authors	Vulnerability indices	Vulnerability scale
		infections.		
Otani 2018	Child health	Conceptualised vulnerability during *dzud* - a term for severe winter, which leads to the loss of lives and livestock.	–	-
Pedraza 2016	Child health	Vulnerability is defined in terms of social vulnerability of families which is related to structural poverty, aggravated by economic problems.	–	-
Pollitt 1996	Child health	The authors took a timing approach to define vulnerability. The idea is that, during periods of rapid brain growth ('brain growth spurt'), the brain is particularly vulnerable to malnutrition.	–	-
Sartorius 2011	Child health	No specific definition of vulnerability was given by the authors.	- Mother's death (especially when due to HIV and tuberculosis)<br>- Greater number of children under 5 years living in the same household.<br>- Winter season.	-
Seidel 2000	Maternal health, child health	The authors defined vulnerability as a situation where a mother is young and unable to afford formula milk to avoid mother-to-child transmission of HIV.	–	-
Siekmans 2014	Child health	The authors defined vulnerability as a concept that encompasses both the external exposure to risk factors and the internal means for coping with those risks without damaging loss. Young children are often considered vulnerable since they are more likely to be harmed by these stressors than others in the general population.	–	-
Ventura 2008	Child health, maternal health	No specific definition of vulnerability was given by the authors.	- Number of people in the household who were working.<br>- Employment situation of the mother and the head of the household at the time of the child's death.<br>- Access to health services: Access to prenatal care.	-
Wang 2019	Child health	No specific definition of vulnerability was given by the authors.	-	Devereux Center for Resilient Children assessment tools (DECA)
Winch	Child	New-born vulnerability was defined in terms of cold	–	

Author & year	Context	Definition by authors	Vulnerability indices	Vulnerability scale
2005	health	air, cold food, or drinks (either directly or indirectly through the mother), and malevolent spirits or evil eye.		
Yuan 2020	Child health	The social vulnerability in this study was defined as the combination of multiple factors including fragile economic foundation, extreme poverty, poor market accessibility, stagnant maternal education level, rapidly inflated population, and serious armed conflict.	- The Poverty and Adaptive Capacity Index (PACI)<br>- Population Exposure Index (PEI)	-
