# Supplementary material for: Vulnerability in maternal, new-born, and child health in low- and middle-income countries: Findings from a scoping review
Source: PLoS One. 2022 Nov 11;17(11):e0276747. doi: 10.1371/journal.pone.0276747 (PMC9651566; doi:10.1371/journal.pone.0276747)
Supplement: S3 Table — (PDF) [file pone.0276747.s003.pdf]

Ranking of suggested definitions of vulnerability in maternal and child health

S/N	Suggested Definitions for Vulnerability in MNCH	Reviewer Number							Average
		1	2	3	4	5	6	7	
1	Women in pregnancy, childbirth or puerperium or children 9 years old or younger who have an increased risk or susceptibility to adverse health outcomes because they experienced limited basic rights or limited resources fulfilled	2	4	4	4	5	3	2	3.0
2	Women in pregnancy, childbirth or puerperium or children 9 years old or younger at greater risk of experiencing physical or emotional and or poor outcomes because of exposure to one or more adverse factors in their lives	1	5	3	2	4	2	3	2.9
3	Women in pregnancy, childbirth or puerperium or children 9 years old or younger that are prone to negative outcomes at a higher rate than their peers due to circumstances beyond their control	3	2	2	3	3	1	4	**2.6**
4	State or condition of women in pregnancy, childbirth or puerperium or children 9 years old or younger who lack health or are susceptible to poor outcomes related to the presence of at least one adverse determinant or lack of access to basic rights and resource	4	1	5	1	1	5	1	**2.6**
5	State or condition of being weak or poorly defended by women in pregnancy, childbirth or puerperium or children 9 years old or younger due to the presence of at least one adverse determinant or lack of access to basic rights and resources	5	3	1	5	2	4	5	3.3

Note: *lower value represents a higher rank while a higher value denotes a higher rank*
