# Supplementary material for: Vulnerability in maternal, new-born, and child health in low- and middle-income countries: Findings from a scoping review
Source: PLoS One. 2022 Nov 11;17(11):e0276747. doi: 10.1371/journal.pone.0276747 (PMC9651566; doi:10.1371/journal.pone.0276747)
Supplement: Attachment — Submitted filename: scoping review_plos one_agost22.docx [file pone.0276747.s005.docx]

This study is a scoping review that follows a systematic review process based on a framework and guidelines similar to Prisma. It has a registered protocol.

The number of databases consulted is sufficient and wide-ranging. The gray literature has been reviewed. However, the date of the last search is old (it took place more than 15 months ago). On the other hand, it is striking that it is in March. I wonder if there is some objective reason for this. I suggest updating the search to at least December 31, 2021 or even later.

The type of review and the methodology used really help to address the objective of the study.

**Minor comments**

This phrase-ending “, with researchers examining their vulnerabilities.” can be omitted I think.

Can you provide a reference for World Bank Country and Lending groups?

Please, provide a reference for Ovid Northern Light Conference Abstracts instead of the description of the text.

Could you please indicate the first and last name acronyms of the authors (within the manuscript) who participated in the screening and extraction phases? What was the inter-rather percentage agreement between the two literature reviewers? Please, add this proportion in the text.

This phrase is repeated and therefore can be omitted > “Two authors independently charted key information from the included Publications”

By curiosity, what is the utility of using Airtable if Excel is also used?

Table 2. Column “Vulnerability scale” could be deleted. This information may be reflected in the text since no study used it to measure vulnerability.

AUTHORS’ CONTRIBUTIONS, COMPETING INTEREST, ACKNOWLEDGEMENT and FUNDING sections are written in different font format.

I missed a flow diagram. It would be convenient to include it.

38 did not meet the inclusion criteria and were subsequently excluded. Please, indicate exactly how the studies were grouped for each exclusion criterion.

How many years did the search period cover? Please, provide this information into the manuscript.

**Language**

This article has been written in an excellent English.
