# Supplementary material for: Vulnerability in maternal, new-born, and child health in low- and middle-income countries: Findings from a scoping review
Source: PLoS One. 2022 Nov 11;17(11):e0276747. doi: 10.1371/journal.pone.0276747 (PMC9651566; doi:10.1371/journal.pone.0276747)
Supplement: Attachment — Submitted filename: PONE_Rebuttal Letter.docx [file pone.0276747.s006.docx]

Dear Editro,

We thank you for the feedback on our work ‘Vulnerability in Maternal, New-born, and Child Health in Low- and Middle-Income Countries: Findings from a scoping review’. We were able to use the feedback from the reviewers to improve the manuscript further. A point by point rebuttal is available below.

We certainly would be available to address any further comments that the reviewers might have.

Kind Regards’

Olusesan Makinde

For the authors.

PONE-D-22-08032

Vulnerability in Maternal, New-born, and Child Health in Low- and Middle-Income Countries: Findings from a scoping review

PLOS ONE

Journal Requirements:

When submitting your revision, we need you to address these additional requirements.

1. Please ensure that your manuscript meets PLOS ONE's style requirements, including those for file naming. The PLOS ONE style templates can be found at 
<https://journals.plos.org/plosone/s/file?id=wjVg/PLOSOne_formatting_sample_main_body.pdf> and 
<https://journals.plos.org/plosone/s/file?id=ba62/PLOSOne_formatting_sample_title_authors_affiliations.pdf>

We have worked on preparing the manuscript in accordance with the style stated at the links.

2. We note that Figures 2 and 3 in your submission contain map images which may be copyrighted. All PLOS content is published under the Creative Commons Attribution License (CC BY 4.0), which means that the manuscript, images, and Supporting Information files will be freely available online, and any third party is permitted to access, download, copy, distribute, and use these materials in any way, even commercially, with proper attribution. For these reasons, we cannot publish previously copyrighted maps or satellite images created using proprietary data, such as Google software (Google Maps, Street View, and Earth). For more information, see our copyright guidelines: <http://journals.plos.org/plosone/s/licenses-and-copyright>.
 
We require you to either (1) present written permission from the copyright holder to publish these figures specifically under the CC BY 4.0 license, or (2) remove the figures from your submission:
 
Response: The maps included in this manuscript were generated by the authors using ‘R’. R is an open-source programming language and there is no copyrighted material included in the entire manuscript.

3. Please include captions for your Supporting Information files at the end of your manuscript, and update any in-text citations to match accordingly. Please see our Supporting Information guidelines for more information: <http://journals.plos.org/plosone/s/supporting-information>.

This has been done.

5. Review Comments to the Author

Please use the space provided to explain your answers to the questions above. You may also include additional comments for the author, including concerns about dual publication, research ethics, or publication ethics. (Please upload your review as an attachment if it exceeds 20,000 characters)

Reviewer #1: Thank you for the opportunity to review this scoping review manuscript. I think this article has the potential to provide new insights into the field of MNCH, particularly in the LMICs context. I do have some inputs to help the authors to improve the manuscript:

I wonder if similar scoping or systematic reviews exist to help elaborate the state of the art and/or research gap in the introduction.

As at the time we started this project, no paper like this had been done. However, we have searched again and did not identify a similar work.

It is common that after conducting a review, a follow-up scan for articles published since articles were gathered is conducted to make sure no new articles had been published in that time. The one-year gap from 2021 to 2022 should be closed by a quick scan. The authors should do this, and if it was done, detail it in the methods section and as a study limitation.

We have updated the paper based on new papers published between our stated search date and August 2022.

What are the credentials of the two reviewers?

Two sets of reviewers who have expertise in maternal and child health independently reviewed the studies for inclusion. The Covidence software provided an opportunity for multiple people to review. In fact, all project authors contributed to this level of effort. Whenever there was a discrepancy, this was addressed during a weekly meeting of all investigators.

Placing the context and settings of the selected articles in the discussion section should improve the analysis. For example, comparing definitions and indices of vulnerability in Africa and comparing them to other regions.

We have added information to improve discussion from the definitions across countries on maternal and child health. However, this was difficult as even within countries, the definitions used by authors was not uniform. So geographic comparison was not that easy.

I think the authors should concentrate on discussing the limitation of the study, instead of the limitation of the method itself.

This section has been improved.

Reviewer #2: This study is a scoping review that follows a systematic review process based on a framework and guidelines similar to Prisma. It has a registered protocol.
The number of databases consulted is sufficient and wide-ranging. The gray literature has been reviewed. However, the date of the last search is old (it took place more than 15 months ago). On the other hand, it is striking that it is in March. I wonder if there is some objective reason for this. I suggest updating the search to at least December 31, 2021 or even later.
The type of review and the methodology used really help to address the objective of the study.

Minor comments:
This phrase-ending “, with researchers examining their vulnerabilities.” can be omitted I think.

This has been addressed.

Can you provide a reference for World Bank Country and Lending groups?

This has been included.

Please, provide a reference for Ovid Northern Light Conference Abstracts instead of the description of the text.

This has been done.

Could you please indicate the first and last name acronyms of the authors (within the manuscript) who participated in the screening and extraction phases?

Due to the Covidence software we used for managing the data, all authors participated in the screening and review of the articles. All authors met once every week to deliberate on any discrepancies till this stage of the research was completed. Thus, a statement mentioning the role all authors played has been further included in this section.

What was the inter-rather percentage agreement between the two literature reviewers? Please, add this proportion in the text.

We didn’t track this proportion during the study.

This phrase is repeated and therefore can be omitted > “Two authors independently charted key information from the included Publications”

This has been edited accordingly

By curiosity, what is the utility of using Airtable if Excel is also used?
Table 2. Column “Vulnerability scale” could be deleted. This information may be reflected in the text since no study used it to measure vulnerability.

It was for convenience and because we were all working remotely, Airtable was a better option when we started with the large dataset.

AUTHORS’ CONTRIBUTIONS, COMPETING INTEREST, ACKNOWLEDGEMENT and FUNDING sections are written in different font format.
I missed a flow diagram. It would be convenient to include it.

It was added as Figure 1.

38 did not meet the inclusion criteria and were subsequently excluded. Please, indicate exactly how the studies were grouped for each exclusion criterion.

We have added information on reasons for exclusion. Due to the update search we did, the number of articles excluded has increased. "Of these, 49 did not meet the inclusion criteria and were subsequently excluded, because of wrong population (n=25), wrong outcomes reported (n=16) and conducted in high-income countries (n=8)."

How many years did the search period cover? Please, provide this information into the manuscript.

There was no limit to the search.
